# TRANS-TACE: Prognostic Role of the Transient Hypertransaminasemia after Conventional Chemoembolization for Hepatocellular Carcinoma

**DOI:** 10.3390/jpm11101041

**Published:** 2021-10-17

**Authors:** Alessandro Granito, Antonio Facciorusso, Rodolfo Sacco, Laura Bartalena, Cristina Mosconi, Ugo Vittorio Cea, Alberta Cappelli, Matteo Antonino, Francesco Modestino, Nicolò Brandi, Francesco Tovoli, Fabio Piscaglia, Rita Golfieri, Matteo Renzulli

**Affiliations:** 1 Division of Internal Medicine, IRCCS Azienda Ospedaliero-Universitaria di Bologna, 40137 Bologna, Italy; alessandro.granito@unibo.it (A.G.); francesco.tovoli2@unibo.it (F.T.); fabio.piscaglia@unibo.it (F.P.); 2 Gastroenterology and Digestive Endoscopy, Department of Medical and Surgical Sciences, University of Foggia, 71100 Foggia, Italy; antonio.facciorusso@virgilio.it (A.F.); r.sacco@ao-pisa.toscana.it (R.S.); ugocea@yahoo.it (U.V.C.); antoninomatteo@gmail.com (M.A.); 3 Department of Radiology, IRCCS Azienda Ospedaliero-Universitaria di Bologna, Via Albertoni 15, 40137 Bologna, Italy; laura.bartalena@studio.unibo.it (L.B.); cristina.mosconi@aosp.bo.it (C.M.); alberta.cappelli@aosp.bo.it (A.C.); francesco.modestino@aosp.bo.it (F.M.); nicolo.brandi@studio.unibo.it (N.B.); rita.golfieri@unibo.it (R.G.)

**Keywords:** lipiodol, hepatocellular carcinoma, chemoembolization, prognostic factors, transaminases

## Abstract

The aim of the present study was to correlate laboratory data and postprocedural parameters after conventional transarterial chemoembolization (cTACE) for hepatocellular carcinoma (HCC) with the radiological response. The study consisted of a retrospective analysis of prospectively collected data from 70 consecutive patients who underwent cTACE. Laboratory parameters were assessed daily after cTACE and compared to pretreatment values. Post-treatment radiological response was assessed using mRECIST at one month from cTACE, and factors associated with treatment response (complete and objective response) were assessed by logistic regression analysis. The optimal cutoff points in predicting the complete response of target lesions were a 52% ALT and a 46% AST increase after cTACE compared to the pre-treatment values. Using multivariate analyses, >46% AST and >52% ALT increases with respect to the pre-treatment value were significantly correlated with the objective response (*p* = 0.03 and *p* = 0.04, respectively) and the complete response (*p* = 0.02 and *p* = 0.02, respectively). No patients experienced liver function deterioration after cTACE, and no specific treatment was required. This study showed that post-treatment transient transaminase elevation was predictive of objective response to superselective cTACE in clinical practice, representing a simple tool to guide treatment strategy of HCC patients in a tailored approach.

## 1. Introduction

In the last decade, hepatocellular carcinoma (HCC), due to its constantly increasing incidence, has become the fifth leading cause of cancer, and one of the most frequent causes of cancer-related mortality worldwide, with a low 5-year survival rate (10–15%) and the most common cause of death in cirrhotic patients [1,2,3,4,5,6,7].

There are several treatment options for HCC; nevertheless, those associated with the higher 5-year survival rate, such as ablation, surgical resection, and liver transplantation, can only be applied in the very early and early stages of the disease [8], accounting for less than 20% of cases at presentation [9]. 

Conversely, the vast majority of cases at presentation are diagnosed at an intermediate and/or advanced stage, and therefore the only treatment options available nowadays are transarterial chemoembolization (TACE), radioembolization (TARE), or systemic therapies [10,11,12,13]. In particular, TACE is the most common treatment option in this clinical setting, with 46.4% initial tumors treated with this technique [14].

The current recommendations from the European Association for the Study of Liver (EASL) state that TACE represents the elective treatment option for patients classified as intermediate stage according to the Barcelona Clinic Liver Cancer staging system (BCLC) [8,15]. Furthermore, it has been demonstrated that TACE can also be applied to the unresectable early stage (BCLC A), and in some cases in advanced-stage patients, often associated with systemic therapy (BCLC C) [16,17]. 

However, cirrhotic patients are very heterogeneous, with different tumor loads, liver function, and disease etiology, suggesting that not all patients will derive a similar benefit from TACE, as can also be seen in the large survival differences reported for individual series [15,18].

Historically, more than 10 factors included in staging systems, such as tumor load and hepatic function, have been proposed for cirrhotic patients, and some of them have been applied in clinical practice in order to predict the natural history and survival in relation to various therapeutic modalities [19,20,21,22,23]. In particular, the BCLC staging system represents the most commonly used score in Western countries to predict prognosis and guide treatments. However, two different studies showed that the prognostic value of BCLC was limited in the setting of TACE [24,25]. 

Therefore, a multitude of TACE-specific staging systems have been developed, such as the hepatoma arterial embolization prognostic (HAP) score [26], the selection for transarterial chemoembolization treatment (STATE) score [27], the Munich-transarterial chemoembolization score (M-TACE) [25], the six-and-twelve score [28], and the albumin–bilirubin (ALBI) grade [29]. These prognostic scores are based on prediction factors collected before the cTACE procedure, such as tumor characteristics (e.g., tumor size, vascular invasion), hepatic function (e.g., albumin, bilirubin blood levels), blood markers such as alpha-fetoprotein (AFP), and chronic liver severity indexes such as the Child–Pugh score [30,31].

However, until now, postprocedural parameters or biomarkers have never been investigated as outcome predictors of TACE’s efficacy. In fact, the treatment response will be demanded by the first post-TACE imaging control, usually performed with computed tomography (CT) or magnetic resonance imaging (MRI). It will be of great clinical benefit to utilize laboratory data, usually performed in the days following the procedure, to assess the hepatic function after cTACE, as well as for a prognostic intent.

The aim of the present study was to correlate laboratory data and postprocedural parameters after conventional TACE (cTACE) with the radiological response, highlighting the possible factors that could predict radiological response, in order to identify a possible post-TACE prognostic score.

## 2. Results

**Figure 1:**
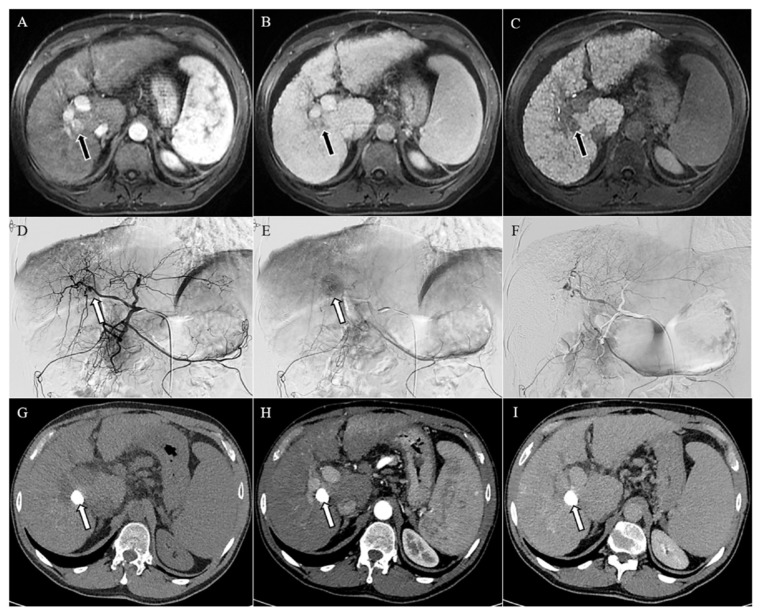
Magnetic resonance imaging (**A**–**C**) showing a lesion of 18 mm in the liver segment 8, hypervascularized in the arterial phase (arrow in (**A**)), with washout of the contrast media in the venous phase (arrow in (**B**)), and hypointense in the hepatobiliary phase (arrow in (**C**)), consistent with hepatocellular carcinoma in the context of cirrhosis. Angiographic study (**D**–**F**) of the liver confirmed the hypervascular lesion in the early and late arterial phases (arrows in (**D**,**E**), respectively), which was no longer visible after chemoembolization (**F**). Computed tomography (**G**–**I**) performed one month after chemoembolization demonstrated an homogeneous accumulation of the lipiodol in the unenhanced image (arrow in (**G**)), with no signs of relapse in the arterial and delayed phases (**H,I**).

The final study population was composed of 70 patients; their demographic, laboratory, and tumoral characteristics are detailed in Table 1 and Figure 1. The vast majority of patients were male (68.5%), with a median age of 69 years (IRQ 61.2–77.7 years); the cirrhotic etiology was HCV infection in half of the cases, and 55.7% of patients were in BCLC stage B. From the perspective of tumor burden, the total number of nodules was 99, HCC was single in 61.5% of patients, and the median maximum diameter was 20 mm. Finally, 80% of patients were allocated within the Milan Criteria.

**Figure 2:**
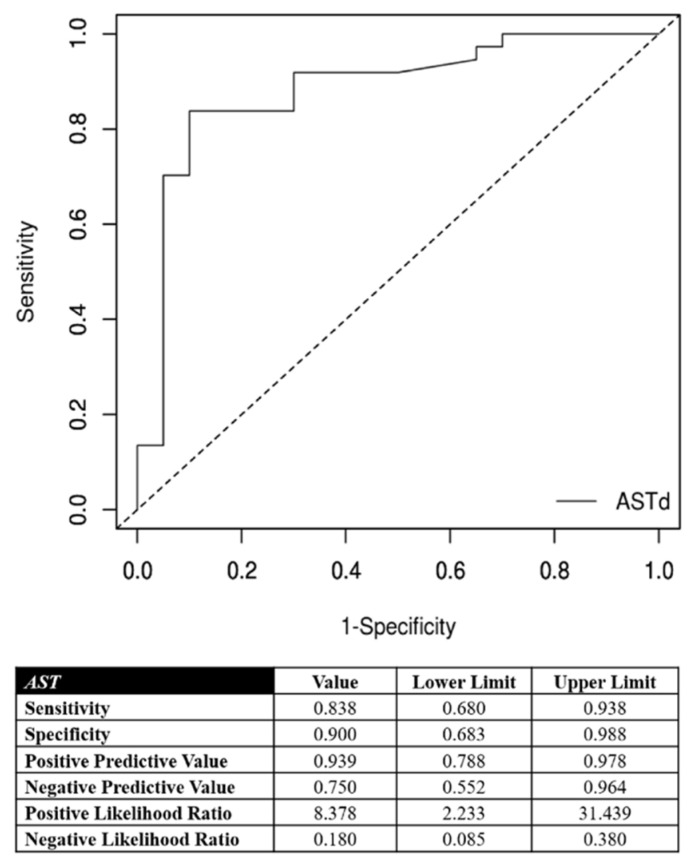
Correlation between aspartate aminotransferase (AST) increase and the objective response for target nodules. In the lower portion of the figure, the performance measures are reported using the optimal cutoff point (46%).

**Figure 3:**
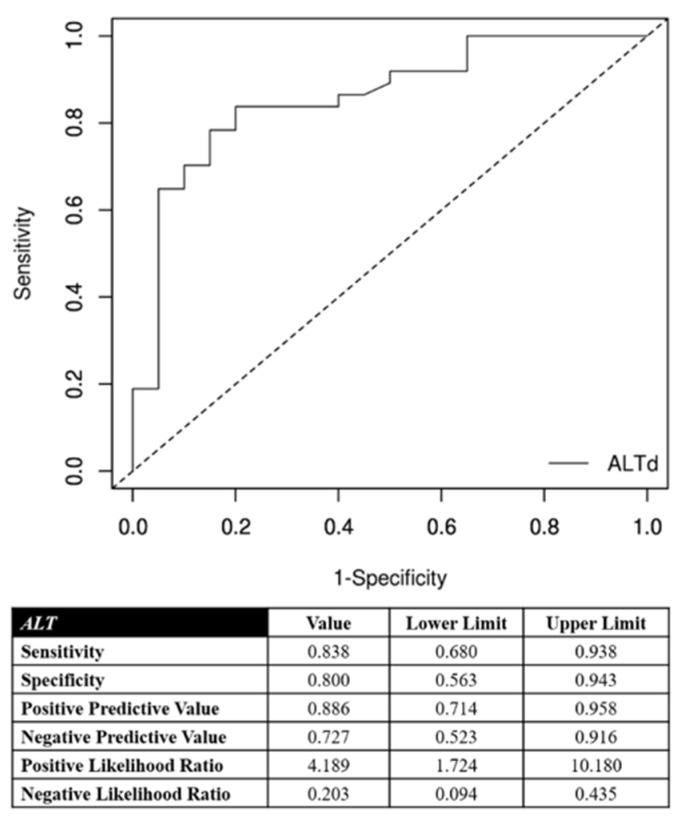
Correlation between alanine aminotransferase (ALT) increase and the objective response for target nodules. In the lower portion of the figure, the performance measures are reported using the optimal cutoff point (52%).

Laboratory data post-cTACE are reported in Table 2. The hospitalization lasted an average of 2.4 days (±0.6 day). The correlation between the AST increase and the objective response of the target nodules are detailed in Figure 2. The optimal cutoff point in predicting objective response of the target lesions was the AST increase of 46% after cTACE procedures with respect to the pretreatment value. This cutoff ensured very accurate diagnostic performance in predicting an objective response of treated nodules and target lesions (Figure 2): its specificity and positive predictive value were 90% and 94%, respectively. Furthermore, the correlation between ALT increase and objective response of the target nodules are detailed in Figure 3. The optimal cutoff point in predicting objective response of the target lesions was the AST increase of 52% after cTACE procedures with respect to the pretreatment values. This cutoff ensured very accurate diagnostic performance in predicting objective response of treated nodules and target lesions (Figure 3): its specificity and positive predictive value were 80% and 89%, respectively.

Univariate and multivariate logistic regression analyses for the assessment of clinical, laboratory, and procedural data associated with objective response and with complete response when considering only the response of the target lesions (lesions treated with cTACE) are detailed in Table 3 and Table 4, respectively. In particular, in the multivariate analyses, among factors regarding patient characteristics, tumor burden, and laboratory data, only AST increase (>46%) and ALT increase (>52%) resulted significantly correlated with the objective (*p* = 0.03 and *p* = 0.04, respectively) and complete (*p* = 0.02 and *p* = 0.02, respectively) response, taking into account only the response of the target lesions. 

Univariate and multivariate logistic regression analyses for the assessment of clinical, laboratory, and procedural data associated with objective response when considering the overall response are detailed in the Table 5. Taking into account the overall response, in the multivariate analysis, among factors related to patient characteristics, tumor burden, and laboratory data, only BCLC B negatively affected the possibility of reaching an objective response.

## 3. Discussion

In this clinical practice study, we evaluated the prognostic significance of changes in liver parameters as predictors of treatment response to cTACE. In a cohort of patients treated with a superselective procedure, a postprocedure increase of transaminases (AST increase ≥46%, ALT increase ≥52%) compared with baseline values was shown to be a reliable predictor of response to cTACE. Furthermore, these transaminase increases were easy to use clinically, because they represented an increase of nearly 50% of the baseline values.

In our opinion, this study featured at least four aspects of clinical relevance. First, this transient increase in serum transaminases was not accompanied by a worsening of the liver functional reserve, suggesting that this effect was mainly secondary to tumor necrosis and not to nontumor liver tissue injury. Previous studies analyzed the prognostic impact of postembolization syndrome (characterized by right upper quadrant abdominal pain, fever, nausea, and vomiting), which was likely caused by arterial embolization also involving normal liver tissue, and reported a lack of association of hypertransaminasemia and postembolization syndrome with improved tumor response [32,33]. 

However, these studies cannot be compared with ours because superselective cTACE was not used. This discrepancy is of great relevance, because the superselective technique allows the embolization to be primarily confined to arteries supplying the tumor, and very limitedly involving normal liver tissue, thus explaining the absent impact on liver function [8,34].

In our patients presenting with transaminase elevation after cTACE, no other concomitant symptoms or liver function deterioration were observed.

Given that transaminases are produced by both hepatocytes and hepatocyte-derived tumor cells, and considering the lack of liver functional reserve deterioration, it is conceivable that the serum concentration elevation of these enzymes was of tumor origin, thus justifying the correlation with tumor response.

Our study was in line with the results of Marquez et al., who demonstrated that the occurrence of hepatic cytolysis (defined as a post-TACE aspartate aminotransferase increase above 100 IU/L with at least doubling of the baseline value) was associated with a favorable radiological response [35]. However, there were many differences between this study and ours. Firstly, the chemotherapeutic agent most often used by Marquez et al. was cisplatin, which, according to the EASL guidelines [8], is not the most effective drug in the treatment of HCC. Furthermore, in the study in [35], different from our experience, the authors did not used the modified RECIST to assess the treatment response, and they performed the imaging control not after one month from the TACE according to the EASL guidelines, but after two months. 

A previous study by Castells et al. hypothesized that hepatic cytolysis associated with fever following transarterial embolization (TAE) represents a clinical marker of tumor necrosis, and therefore, a favorable response to treatment [36]. However, this study differed from ours not only in the different intra-arterial treatment used (TAE), but also in the technique used. In fact, the author reported that the level of occlusion was the main hepatic trunk in 59% of cases, and at the level of the right or left branch in the remaining, and therefore without a superselective approach.

Therefore, our study evaluated for the first time the prognostic impact of hepatic cytolysis after superselective cTACE, the efficacy of which was established using the radiological criteria recommended by current guidelines, unlike previous studies that aimed to assess this prognostic correlation.

A further aspect of clinical relevance that therefore deserves to be emphasized is that transaminase elevation after TACE did not require specific therapeutic interventions beyond antipyretics such as paracetamol. In patients who underwent cTACE according to current international guidelines, it resolved within few days, thus allowing them to avoid an extended hospitalization. We also confirmed the previous results of Castells et al. [36], according to which the occurrence of fever associated with hepatic cytolysis after TACE, being secondary to tumor necrosis, did not require antibiotic therapy.

Another aspect of our study that deserves to be highlighted is that our patients had a median nodule diameter of 20 mm, and this could most likely at least partially justify an embolization limited to only the arterial branches afferent to the tumor nodule. Therefore, our results should be verified in larger nodules that, according to current guidelines, are amenable to treatment with TACE. Even large lesions most likely will not require extensive embolization of healthy liver parenchyma by using a superselective technique, but this will be a subject of future research. Furthermore, in our opinion, it is of great interest that, in line with previously reported clinical practice data, a significant percentage of BCLC stage A and “Milan in” patients could not be treated with curative treatments due to contraindications or suboptimal ultrasound visualization, and after being treated with cTACE, presented response and recurrence rates comparable to those obtained with ablation and surgical resection. Therefore, this study confirmed that improved outcomes were achieved with novel superselective techniques, which can be employed for diverse applications ranging from curative-intent for small tumors to downstaging or bridging to resection and transplantation for early and intermediate BCLC stages [37]. 

A final clinical implication of the present study was that our prognostic results could also be used as a guide to choose the best imaging technique to adopt for the one-month follow-up. Taking into account the different peculiarities of the imaging methods (CT and MRI) recommended by the EASL guidelines to assess the treatment response [8], it would be possible to tailor the best imaging modality case by case. In fact, in the absence of transient transaminase elevation (the probability of recurrence may be higher), it will be preferable to perform MRI instead of CT due to its better diagnostic accuracy in the assessment of the post-cTACE response. Furthermore, starting from a management point of view, a test that predicts the chance of response (or not) allows planning the one-month clinical/radiological management.

Our study had some limitations. Firstly, this was a small series of patients treated with cTACE. However, this one-year series came from a single hepatology unit to avoid the bias of enrolling patients from different clinical units that could adopt different treatment strategies. Moreover, the treatment response to cTACE was equally assessed by CT or MRI. However, our approach was in line with the EASL recommendations.

Further, we did not assess the overall survival. However, our study was not aimed to investigate such endpoint. Lastly, we enrolled only naïve patients, but this strategy was chosen to avoid bias in the assessment of the transient hypertransaminasemia owing to potential functional hepatic reserve deterioration due to previous treatments.

## 4. Materials and Methods

The local institutional review board approved this prospective study, and written informed consent was obtained from all the patients. This study was conducted according to the Declaration of Helsinki for clinical studies.

All the patients who underwent cTACE from 1 February 2017 to 31 December 2018 at our institution were enrolled. The inclusion criteria were naïve patients who underwent cTACE according to the EASL guidelines [8]. Therefore, patients who had previously undergone cTACE were excluded. 

All the cTACE procedures were performed by interventional radiologists with more than 15 years of experience in cTACE. The angiographic procedures were performed as described in our previous papers [38,39]. In particular, after microcatheter placement in tumor-feeding vessels, a mixture of 10 mL of standard iodized oil (Lipiodol^®^; Guerbet, Milan, Italy) and a 50 mg of epirubicin powder (Farmorubicin; Pfizer, Latina, Italy), manually shaken, was injected under fluoroscopic control, followed by embolization with Spongel (Spongostan, Ferrosan Medical Devices A/S, Søborg, Denmark) particles until there was complete stasis in the vessels. 

Different tumor burden and cTACE parameters were evaluated and recorded on a dedicated database:

1. Number of nodules treated;
2. Nodule location, reported as right lobe, left lobe, caudate lobe, or bilateral;
3. Number of embolized segmental arteries.

For each nodule treated, we also collected:

- Size, measured as the maximum diameter of the lesion expressed in mm;
- Location, recorded as liver segments from 1 to 8;
- Nodule site: peripheral or central;
- Type of vascularization, recorded as intrahepatic or extrahepatic.

Radiological tumor response was assessed either by CT or MRI one month after the procedure according to the Modified RECIST assessment for HCC [40]. 

Laboratory tests carried out one day before cTACE and in the following days after procedure, comprehensive of hepatic function panel (i.e., alanine transaminase, aspartate transaminase, bilirubin, albumin, alkaline phosphatase, and gamma-glutamyl transferase), were evaluated and recorded in the dedicated database. Specifically, the maximum value reached by the abovementioned parameters in the days following cTACE were collected. Postprocedural parameters, such as postembolization syndrome or ascites after the procedure, were recorded as well, and grades of symptoms were recorded according to the Common Terminology Criteria for Adverse Events (CTCAE) v5.0. 

### Statistical Analysis

Categorical variables were described as frequencies and percentages, and continuous variables as median and interquartile range (IQR).

The inferential analysis for objective and complete response was conducted using the Cox univariate and multivariate logistic regression model to estimate odds ratios (ORs) and 95% confidence intervals (CIs). In this analysis, cutoff values for AST and ALT increases, evaluated as a percentual growth respect to the basal value, were computed through receiver operating characteristics (ROC) curves. Statistically significant variables from the univariate Cox analysis were consistently included in the multivariate model. Different Cox models were built for the analysis of target lesions and the overall response.

The analysis was performed using R Statistical Software (Foundation for Statistical Computing, Vienna, Austria), and significance was established at the 0.05 level (two-sided).

## 5. Conclusions

In conclusion, this study demonstrated that transaminase elevation after superselective cTACE was a simple and accurate clinical marker that predicted treatment response to cTACE, and identified patients in whom, instead, tumor nodules/vascularization had a low likelihood of response, for which it therefore may be more beneficial to consider alternative treatments. Hypertransaminasemia after superselective cTACE was a transient event, and therefore, in well-selected patients, was not associated with liver function deterioration that would compromise the chance of repeated/sequential intra-arterial treatments or systemic therapies.

## Figures and Tables

**Table 1:** Demographical characteristics and tumor burden of the study population at baseline.

	Patients (n = 70) Median (IQR) or N. (%)
Age (years)	69 (61.2–77.7)
Gender (M/F)	48 (68.5)/22 (31.5)
Milan in	56 (80)
Alcohol abuse	8 (11.4)
Etiology (HCV/HBV/other)	35 (50)/6 (8.5)/29 (41.5)
AST (n.v. < 35 U/L)	47 (33–80.7)
ALT (n.v. < 35 U/L)	31.5 (23.25–52.7)
GGT (n.v. < 38 U/L)	62.5 (35–112.5)
Alkaline phosphatase (n.v. 30–120 U/L)	107.5 (80.7–144.7)
Total bilirubin (n.v. < 1.2 mg/dL)	1.28 (0.79–1.64)
AFP (n.v. < 10 ng/mL)	5.85 (3.4–18.8)
Child–Pugh Score (A/B7)	59 (84.3)/11 (15.7)
Nodules per patient	1 (1–2)
Number of nodules per patient: 1 2 3	43 (61.5) 25 (35.7) 2 (2.8)
Max diameter (mm)	20 (15.25–27.25)
Lobe (right/left/caudate/bilobar)	47 (67.1)/12 (17.1)/2 (2.8)/9 (13)
BCLC (A/B)	31 (44.3)/39 (55.7)

IQR, interquartile range; HCV, hepatitis C virus; HBV, hepatitis B virus; n.v., normal value; AST, aspartate aminotransferase; ALT, alanine aminotransferase; GGT, gamma-glutamyl transferase; AFP, alpha-fetoprotein; BCLC, Barcelona Cancer of the Liver Clinic.

**Table 2:** Laboratory data after conventional chemoembolization.

	Patients (*n* = 70) Median (IQR) or N. (%)
AST (n.v. < 35 U/L)	150.5 (55–240.8)
ALT (n.v. < 35 U/L)	122 (38.2–231.7)
AST increase	67% (7–488%)
ALT increase	96% (7–572%)
GGT (n.v. < 38 U/L)	61 (32.2–122.2)
Alkaline phosphatase (n.v. 30–120 U/L)	101 (78–138)
Total bilirubin (n.v. < 1.2 mg/dL)	1.52 (0.94–1.86)
Postembolization ascites	7 (10)
Child–Pugh Score (A/B)	53 (75.7)/17 (24.3)
Postembolization syndrome	22 (31.4)

IQR, interquartile range; n.v., normal value; AST, aspartate aminotransferase; ALT, alanine aminotransferase; GGT, gamma-glutamyl transferase.

**Table 3:** Univariate and multivariate logistic regression analyses for the assessment of clinical, laboratory, and procedural data associated with objective response when considering only the response of the target lesions (lesions treated with conventional chemoembolization).

	Univariate Analysis (OR CI 95%)	*p*	Multivariate Analysis (OR CI 95%)	*p*
Age (reference ≤ 65 years)	1.12 (0.86–2.48)	0.16		
Gender (reference F)	1.06 (0.78–1.3)	0.58		
Etiology (reference HCV)	HBV: 0.77 (0.61–1.41) Other: 1.14 (0.77–3.09)	0.3 0.13		
Child–Pugh (reference A)	1.39 (0.88–2.11)	0.25		
Milan in (no reference)	0.57 (0.15–1.73)	0.43		
AST (ref ≤ 47)	1.004 (0.99–1.01)	0.41		
ALT (reference ≤ 31.5)	1.00 (0.99–1.01)	0.87		
AFP (reference ≤ 20 UI/mL)	1.04 (0.93–1.11)	0.43		
GGT (reference ≤ 62.5)	0.99 (0.45–1.47)	0.45		
Number of treated arteries	1.55 (0.40–3.93)	0.44		
Max diameter (reference ≤ 20 mm)	1.15 (1.08–1.26)	0.001	1.03 (0.91–1.17)	0.15
Number of nodules (reference 1)	0.78 (0.52–0.92)	0.05	0.79 (0.65–2.51)	0.23
BCLC (reference A)	0.48 (0.28–1.17)	0.14		
Post-TACE ascites (no reference)	3.11 (1.8–5.3)	<0.001	3.57 (0.73–5.2)	0.35
Fever (no reference)	2.76 (0.92–7.55)	0.15		
AST increase (reference ≤ 46%)	1.91 (1.37–3.13)	0.007	1.15 (1.10–2.89)	0.03
ALT increase (reference ≤ 52%)	1.66 (1.28–2.42)	0.006	1.40 (1.21–2.69)	0.04

OR, odds ratio; AST, aspartate aminotransferase; ALT, alanine aminotransferase; GGT, gamma-glutamyl transferase; AFP, alpha-fetoprotein; BCLC, Barcelona Cancer of the Liver Clinic; TACE, transarterial chemoembolization.

**Table 4:** Univariate and multivariate logistic regression analyses for the assessment of clinical, laboratory, and procedural data associated with the complete response of the target lesions.

	Univariate Analysis (OR CI 95%)	*p*	Multivariate Analysis (OR CI 95%)	*p*
Age (reference ≤ 65 years)	1.15 (0.76–1.84)	0.33		
Gender (reference F)	0.98 (0.75–1.41)	0.44		
Etiology (reference HCV)	HBV: 0.67 (0.52–1.29) Other: 1.19 (0.71–4.15)	0.4 0.24		
Child–Pugh (reference A)	1.18 (0.78–2.18)	0.43		
Milan in (no reference)	0.59 (0.11–1.13)	0.39		
AST (reference ≤ 47 U/L)	1.21 (0.75–1.41)	0.11		
ALT (reference ≤ 31.5 U/L)	1.09 (0.92–1.41)	0.39		
AFP (reference ≤ 20 ng/mL)	1.15 (0.69–1.71)	0.34		
GGT (reference ≤ 62.5 U/L)	0.89 (0.58–1.27)	0.71		
Number of arteries	1.39 (0.41–2.39)	0.39		
Max diameter (reference ≤ 20 mm)	1.02 (0.78–1.32)	0.31		
Number of nodules (reference 1)	0.48 (0.32–0.75)	0.03	0.59 (0.45–1.51)	0.35
BCLC (reference A)	0.65 (0.48–2.21)	0.34		
Post-TACE ascites (no reference)	2.38 (1.1–3.2)	0.009	1.87 (0.86–2.5)	0.15
Fever (no reference)	2.14 (0.58–4.51)	0.09		
AST increase (reference ≤ 46%)	2.01 (1.45–3.73)	0.003	1.84 (1.17–2.98)	0.02
ALT increase (reference ≤ 52%)	1.52 (1.19–2.22)	0.004	1.42 (1.18–2.75)	0.02

OR, odds ratio; AST, aspartate aminotransferase; ALT, alanine aminotransferase; GGT, gamma-glutamyl transferase; AFP, alpha-fetoprotein; BCLC, Barcelona Cancer of the Liver Clinic; TACE, transarterial chemoembolization.

**Table 5:** Univariate and multivariate logistic regression analyses for the assessment of clinical, laboratory, and procedural data associated with the objective response when considering the overall response.

	Univariate Analysis (OR CI 95%)	*p*	Multivariate Analysis (OR CI 95%)	*p*
Age (reference ≤ 65 years)	1.24 (0.81–2.23)	0.22		
Gender (reference F)	1.12 (0.65–1.41)	0.38		
Etiology (reference HCV)	HBV: 1.10 (0.41–2.41) Other: 1.34 (0.72–1.89)	0.5 0.21		
Child–Pugh (reference A)	0.98 (0.58–2.17)	0.29		
Milan in (no reference)	0.58 (0.22–1.43)	0.26		
AST (reference ≤ 47 U/L)	1.04 (0.95–1.01)	0.41		
ALT (reference ≤ 31.5 U/L)	1.03 (0.91–1.09)	0.67		
AFP (reference ≤ 20 UI/mL)	0.99 (0.95–1.21)	0.34		
GGT (reference ≤ 62.5 U/L)	1.12 (0.45–2.27)	0.34		
Number of treated arteries	1.93 (0.91–4.5)	0.14		
Max diameter (reference ≤ 20 mm)	1.09 (1.04–1.70)	0.009	1.03 (0.88–1.53)	0.23
Number of nodules (reference 1)	0.86 (0.52–0.91)	0.04	0.84 (0.75–1.15)	0.28
BCLC (reference A)	0.51 (0.29–0.88)	0.03	0.48 (0.33–0.92)	0.04
Post-TACE ascites (no reference)	2.52 (1.1–3.53)	0.01	1.33 (0.83–4.2)	0.28
Fever (no reference)	2.76 (0.92–7.55)	0.15		
AST increase (reference ≤ 46%)	1.05 (0.98–1.15)	0.23		
ALT increase (reference ≤ 52%)	1.05 (0.95–1.18)	0.40		

OR, odds ratio; AST, aspartate aminotransferase; ALT, alanine aminotransferase; GGT, gamma-glutamyl transferase; AFP, alpha-fetoprotein; BCLC, Barcelona Cancer of the Liver Clinic; TACE, transarterial chemoembolization.

## Data Availability

The data presented in this study are available upon request from the corresponding author.
